# Public knowledge, awareness, and practices regarding rabies in Palestine: a cross-sectional survey, 2025

**DOI:** 10.3389/fpubh.2025.1737766

**Published:** 2025-12-18

**Authors:** Akram Amro, Alhareth M. Amro, Anas K. Assi, Salahaldeen Deeb, Amro Odeh, Habeeb H. Awwad, Yahya Kayed AbuJwaid

**Affiliations:** 1 Faculty of Health Professions, Al-Quds University, Jerusalem, Palestine; 2 Faculty of Medicine, Al-Quds University, Jerusalem, Palestine

**Keywords:** post-exposure prophylaxis, practices, preventive measures, public health, rabies, vaccine, veterinary services

## Abstract

**Background:**

Rabies remains a major public health concern in many parts of the world, including Palestine. Although preventable through vaccination and timely post-exposure prophylaxis (PEP), rabies continues to cause preventable deaths, particularly in disadvantaged populations. This study aims to assess public knowledge, awareness, and practices related to rabies prevention in Palestine.

**Methods:**

A cross-sectional survey was conducted from March to August 2025, involving 417 Palestinian adults (≥18 years) from urban and rural areas. A structured, self-administered, pre-validated questionnaire was used to assess awareness, knowledge, and practices related to rabies prevention. Descriptive statistics and multivariable logistic regression were used to analyze the data.

**Results:**

While 89.0% of respondents had heard of rabies, only 42.3% achieved satisfactory knowledge (≥50% correct answers), with significant gaps in understanding the viral etiology and preventive measures. The majority (83.2%) reported they would seek medical care following a dog bite; however, only 11.8% identified wound washing with soap and water as a key first-aid measure. A substantial portion of respondents (67.9%) perceived local veterinary services as inadequate. Knowledge was significantly associated with education level and age, with higher education correlating with better knowledge, while older adults had lower knowledge scores.

**Conclusion:**

Despite high awareness of rabies, critical gaps in knowledge and practices remain in Palestine, particularly concerning transmission routes, prevention, and first-aid measures. Tailored educational campaigns are needed to address these gaps, focusing on the importance of timely wound care and PEP. Additionally, strengthening veterinary services and integrating a One Health approach will be essential to improve rabies prevention and control in Palestine.

## Introduction

Rabies is a long-recognized zoonotic disease and continues to be among the most fatal infectious threats globally, with dog-mediated transmission responsible for the overwhelming majority of human cases (1, 2). Despite its preventability through mass dog vaccination and timely post-exposure prophylaxis (PEP), rabies remains a significant public health challenge in many countries, particularly where veterinary services are limited or fragmented (3, 4). Studies from endemic regions consistently highlight persistent gaps in public awareness regarding transmission routes, symptoms, and preventive actions (5–7), underscoring the continued need for strengthened health education and coordinated One Health approaches.

Across the broader Middle East and North Africa (MENA) region, dog-mediated rabies persists as a public health concern. Evidence from nearby settings demonstrates that gaps in the knowledge, underreporting of exposures, and limited access to veterinary services contribute to ongoing transmission risks (5, 7). Structural challenges such as weak surveillance systems, insufficient vaccination coverage, and inconsistent municipal engagement in dog population management mirror the constraints described in other low-resource or transitional health systems (3, 4). These contextual parallels are particularly relevant for Palestine, where public health planning frequently operates under resource limitations and where municipal and veterinary services may vary considerably across districts.

At present, no formal national rabies surveillance system exists in Palestine, and publicly available data on dog-bite incidence, confirmed human rabies cases, and dog vaccination coverage are not systematically reported; WHO and OIE/WOAH regional reports similarly indicate an absence of consolidated national statistics (8). Despite these limitations, available epidemiological fragments underscore that rabies constitutes an ongoing public health concern. A serological investigation of stray dogs by Fayyad et al. (9) reported that only 11.95% (11/92) of sampled animals from seven Palestinian districts had protective antirabies antibody titres ≥0.5 IU/mL, indicating that the vast majority of free-roaming dogs are immunologically susceptible and capable of sustaining transmission. This study, which represents the first systematic detection of rabies virus–specific antibodies in stray dogs in Palestine, highlighted the high potential for rabies circulation in poorly vaccinated dog populations. Historically, Shimshony (10) have described the persistence of rabies in the broader region, noting that control efforts are complicated by wildlife reservoirs and cross-border animal movement, underscoring that rabies is a transboundary zoonotic threat in this part of the Middle East. Taken together, these consistent lines of evidence indicate that, despite incomplete formal surveillance, Palestine remains vulnerable to dog-mediated rabies. Under such conditions, public reliance on personal knowledge, awareness, and intended practices becomes an important determinant of timely care-seeking behavior and overall rabies risk reduction, reinforcing the need to characterize community-level rabies knowledge and practices in this setting.

Assessment of knowledge, awareness, and practices provides a critical framework for identifying misconceptions, understanding behavioral drivers, and informing community-level interventions. Prior work in other endemic regions has shown that knowledge, awareness, and practices surveys are highly effective in shaping public health strategies, improving dog vaccination uptake, and reducing delays in PEP following exposure (7, 11). In the Palestinian context where structured rabies surveillance and large-scale preventive programs face operational barriers evaluating knowledge, awareness, and practices is essential to guide both health education efforts and municipal-level control measures.

To date, no comprehensive population-based assessment of rabies-related knowledge, awareness, and practices has been conducted in Palestine. Therefore, this study aims to evaluate public knowledge, awareness, and practices regarding rabies prevention among Palestinian adults. The findings seek to inform policy development, strengthen public health messaging, and support integrated One Health approaches to rabies control in Palestine.

Rabies continues to pose a significant public health concern in Palestine, with sporadic reports of suspected rabid animals, frequent dog-bite incidents, and recurring challenges in implementing consistent dog vaccination programs. Limited veterinary infrastructure, fragmented animal-health surveillance, and movement restrictions between districts hinder timely reporting and coordinated response efforts. Additionally, public health initiatives targeting zoonotic diseases often receive limited prioritization due to competing healthcare burdens and resource constraints. These contextual factors heighten the potential for delayed care-seeking, underreporting of exposures, and persistence of misconceptions regarding rabies transmission and prevention. Given these system-level vulnerabilities, understanding knowledge, perceptions, and intended practices is essential to informing targeted educational interventions and strengthening rabies prevention strategies in Palestine.

## Methodology

### Study design and setting

A web-based cross-sectional survey was conducted among residents of Palestine from March 2025 to August 2025 to assess rabies awareness, knowledge, and related practices. The study setting is characterized by a heterogeneous landscape comprising urban cities, rural villages, and refugee camps, each with varying levels of infrastructure and municipal capacity, particularly regarding veterinary services. Due to logistical constraints and the geographically fragmented nature of Palestine, a non-probability snowball sampling strategy was employed. This approach was used for feasibility rather than representativeness and relied entirely on online dissemination. A structured, pre-validated questionnaire was adapted from Al-Mustapha et al. (12).

The survey link was disseminated using digital snowball sampling through university networks, community organizations, local social media groups, health-awareness forums, and municipality pages. Participants were encouraged to share the survey within their personal and community networks. While this approach enabled the survey to circulate across different governorates, it reached only individuals with stable internet access and active online engagement, and therefore should be understood as a convenience sample of digitally connected respondents, rather than coverage of the broader population. Because household visits and offline recruitment methods were not feasible, the online method did not ensure inclusion of individuals without internet access particularly older adults, residents of remote rural areas, and lower-income groups. Self-reported residence categories (urban, rural, and camp) were collected for descriptive purposes only and should not be interpreted as evidence of population-level representation.

The questionnaire was reviewed for content and face validity by multiple experts to ensure clarity and functionality. It was self-administered in Arabic and hosted online via Google Forms, distributed exclusively through social media platforms (e.g., Facebook, WhatsApp). The survey link was initially distributed by the research team to “seed” participants across different governorates, who then shared it within their networks. Because this non-probability digital sampling design inherently excludes individuals without adequate internet access, the sample reflects only those reachable through online referral chains. Participation was voluntary and anonymous, with no incentives offered, and the study does not claim representativeness of the general Palestinian population.

### Participants

A total of 417 respondents participated in the study. The target population comprised adult Palestinian residents (≥18 years). Eligibility was determined based on self-reported demographic data provided in the initial screening section of the questionnaire. Inclusion criteria required participants to be residents of Palestine, aged 18 years or older, and capable of reading Arabic. To minimize bias arising from specialized professional knowledge, we explicitly excluded healthcare professionals (human and veterinary) and students in these fields. While the study aimed to recruit from diverse localities, we acknowledge that the reliance on online sampling creates a “digital divide” bias. Consequently, even among respondents from rural areas, the sample likely over-represents those with higher literacy and socioeconomic status, while individuals without internet access who may be at varying risks for rabies exposure are effectively excluded. As this was an anonymous web-based survey, independent verification of age, residency, and professional status was not feasible; thus, eligibility relied entirely on the honesty of respondents’ self-declarations.

### Sample size calculation

Prior to data collection, we estimated a baseline sample size using standard formulas (Raosoft® sample size calculator) with a 95% confidence level and a 5% margin of error. Since this is the first study to assess rabies knowledge in Palestine, no prior prevalence data were available; therefore, we used a conservative response distribution of 50% to maximize variance and yield the largest required sample size. This calculation resulted in a minimum target of 377 respondents. However, we acknowledge that this formula assumes probability-based simple random sampling, whereas this study employed a non-probability snowball recruitment strategy. Consequently, the calculated sample size served as an approximate operational target to ensure sufficient data volume for analysis, rather than a definitive threshold for statistical power or generalizability. We further recognize that while obtaining a sample size (n = 417) larger than the calculated target improves the precision of estimates within the sampled group, it does not correct the systematic selection bias inherent to the non-random sampling design.

### Questionnaire instrument

The questionnaire was adapted from a previously published survey on rabies in Nigeria (12). It was translated into Arabic by bilingual experts and back-translated to ensure linguistic validity. The survey instrument was focused exclusively on assessing awareness, knowledge, and practices. Attitudes were not measured in this study. To ensure the instrument’s suitability for the Palestinian context, the Arabic version was pilot-tested among 15 adults from the target population to assess clarity, flow, and comprehensibility. Feedback from this pilot led to minor refinements in wording, and the final version was approved by an expert panel prior to data collection. The questionnaire’s awareness section asked respondents if they had ever heard of rabies. Those answering “yes” moved on to the knowledge section, which included items on the cause of rabies, its symptoms, modes of transmission, and prevention measures. Each knowledge item was multiple-choice or multiple-response with correct answers predetermined based on scientific fact. The practices section covered topics such as intended actions after a dog bite (e.g., washing wound, seeking medical care), dog confinement habits (e.g., tethering indoors), willingness to have a veterinarian vaccinate one’s dog, typical dog feeding and care practices, sources of dogs (e.g., buying, adoption), and how dogs are managed when they can no longer be kept. Given the low prevalence of dog ownership in the study population (4.8%), restricting practice questions to current owners would have yielded an insufficient sample size for analysis. Therefore, hypothetical scenarios (e.g., “if you owned a dog…”) were utilized to assess the latent preparedness of the broader community. We acknowledge that these hypothetical responses reflect intended behaviors rather than actual observed practices and may be subject to social desirability bias. Demographic items used standard categorical formats (e.g., age in ranges, education level). The questionnaire structure and content closely followed the original Nigerian study, with any necessary cultural adaptations. The internal consistency of the knowledge items was assessed using Cronbach’s alpha coefficient, which was calculated at 0.728, indicating satisfactory reliability for the study population. Unlike the knowledge scale, practice items were analyzed individually using frequencies and percentages rather than as a composite scale score. This analytical approach was chosen because the items represent conceptually distinct and independent behaviors that do not measure a single underlying construct. Calculating internal consistency reliability coefficients like Cronbach’s alpham would be inappropriate for such heterogeneous behavioral items, as low inter-item correlations reflect the multidimensional nature of the practices rather than poor measurement quality.

### Data analysis

Completed survey data were downloaded from Google Forms and exported into SPSS (version 26) for analysis. Completed questionnaires were coded and entered into a database for analysis. Descriptive statistics summarized the sample: frequencies and percentages for categorical variables (e.g., gender, education, residence) and means (±SD) for continuous measures. Knowledge responses were scored by assigning one point for each correct answer and only those who had heard of rabies contributed to the knowledge score calculation. A composite knowledge score was computed by summing all correct responses (across cause, symptoms, transmission, prevention, and control questions) for each respondent. Scores were converted to a percentage of the maximum possible; consistent with prior studies, we classified scores ≥50% as “satisfactory” knowledge. This threshold was chosen to establish a baseline of fundamental awareness necessary for rabies prevention, this threshold represents a minimum competency level, indicating that respondents correctly answered at least half of the knowledge items, and has been validated in similar survey-based assessments of rabies knowledge in endemic settings (11, 12). We report the mean knowledge score (and SD) and the proportion of respondents with satisfactory knowledge. Practices items were analyzed via frequencies (e.g., percentage who own dogs, who would take a bite victim to the hospital, etc.).

To identify factors associated with satisfactory rabies knowledge, multivariable logistic regression analysis was performed among the 371 respondents who had heard of rabies. Socio-demographic variables were recoded for analysis: age was grouped into four categories (18–25, 26–35, 36–45, ≥46 years), education into low, medium, and high, employment into employed, student, and not working, locality into city versus village/camp, and household economic status into low, average, and high. Knowledge scores were dichotomized at 50% (≥7.5 out of 15) to classify knowledge as satisfactory versus unsatisfactory, consistent with established conventions in health knowledge assessment research. Prior to analysis, logistic regression assumptions were systematically assessed. Variance Inflation Factors (VIF) for all predictors ranged from 1.10 to 2.35, all well below the acceptable threshold of 10, indicating minimal multicollinearity. Model fit was evaluated using the Hosmer-Lemeshow goodness-of-fit test, which yielded χ^2^ = 8.06 (df = 8, *p* = 0.427), indicating adequate calibration, with an area under the ROC curve (AUC) of 0.649 reflecting modest but acceptable discrimination. Sample size sufficiency was verified with at least 10 outcome events per predictor variable. Univariate logistic regression was performed for each predictor, and variables were then entered simultaneously into a multivariate model to estimate adjusted odds ratios with 95% confidence intervals. Statistical significance was set at *α* = 0.05 using two-sided tests. However, given the non-probability snowball sampling design, we explicitly acknowledge that these inferential statistics (*p*-values and confidence intervals) cannot be generalized to the wider Palestinian population. Instead, they should be interpreted descriptively as exploratory associations within this specific convenience sample.

Handling of Missing Data: Prior to analysis, data were screened for missing values. Given the online nature of the survey, responses were mandatory for all Rabies-related items, minimizing missing data. However, for demographic variables, any cases with >10% missing responses were excluded from the final analysis. For cases with ≤10% missing values, mean imputation was applied to maintain statistical power.

### Ethical considerations

The study protocol was reviewed and approved by the Institutional Review Board of Al-Quds University. All procedures conformed to the ethical standards of the Declaration of Helsinki. Before beginning the survey, participants were presented with an online informed-consent statement explaining the objectives, procedures, voluntary nature of participation, and confidentiality measures. Only those who consented were allowed to proceed. Data were collected anonymously; no personally identifying information was obtained. Participants were informed that they could withdraw at any time without penalty. Survey data were stored securely and accessed only by the research team.

## Results

### Socio-demographic characteristics

A total of 417 respondents completed the survey. Reflecting the nature of the online recruitment strategy, the sample exhibited a substantial demographic skew toward younger and more educated individuals. 36.7% were aged 18–25 years and 22.5% were 26–35 years. The sample was 55.4% female. Participants resided mainly in Hebron (32.9%), Salfit (19.7%), and Ramallah/Al-Bireh (17.3%), with smaller proportions from other areas; Over half of the respondents lived in urban areas (52.3%), 44.8% in rural communities, and 2.9% in refugee camps. Educational attainment was high: 62.1% held a university bachelor’s degree and 10.8% had postgraduate education, whereas only 1.0% had no formal education. Over half were employed (56.6%), with students and housewives comprising 19.2 and 10.8%, respectively. Most reported an average household income (68.8%), with 19.7% low income and 11.5% high income. We acknowledge that this overrepresentation of digitally connected respondents likely inflates the reported awareness and knowledge scores compared to the general population and may influence the strength of the observed regression associations. (Table 1).

**Table 1:** Socio-demographic characteristics of respondents (*N* = 417).

Variable	Category	*n*	%
Age	18–25	153	36.7
	26–35	94	22.5
	36–45	86	20.6
	46–55	53	12.7
	56–65	26	6.2
	More than 65	5	1.2
Gender	Male	186	44.6
	Female	231	55.4
Area of residence	Ramallah and Al-Bireh	72	17.3
	Hebron	137	32.9
	Bethlehem	21	5.0
	Nablus	76	18.2
	Jerusalem	8	1.9
	Jenin	10	2.4
	Tulkarm	6	1.4
	Tubas	1	0.2
	Salfit	82	19.7
	Qalqilya	2	0.5
	Palestinian inside the 1948 territories	2	0.5
Locality type	City	218	52.3
	Village	187	44.8
	Refugee Camp	12	2.9
Highest level of education	No formal education	4	1.0
	Primary education	12	2.9
	Secondary education	66	15.8
	Diploma/Community College	31	7.4
	University Degree (Bachelor’s)	259	62.1
	Postgraduate (Master/PhD)	45	10.8
Employment status	Employed	236	56.6
	Unemployed	44	10.6
	Student	80	19.2
	Housewife	45	10.8
	Retired	12	2.9
Household’s economic situation	Low income	82	19.7
	Average income	287	68.8
	High income	48	11.5

### Awareness and knowledge of canine rabies

Most respondents (89.0%) reported having heard of rabies. These 371 respondents completed the knowledge assessment. The knowledge scale consisted of 15 items covering causes, symptoms, transmission, and prevention, with a possible score range of 0 to 15. The overall mean knowledge score was 6.71 (SD 3.54), corresponding to a mean knowledge percentage of 44.7%. Using the pre-defined cut-off of ≥50% to indicate a minimum competency level, fewer than half (42.3%) achieved a “satisfactory” knowledge level (≥50% correct). Only 56.9% correctly identified a virus as the cause of rabies, but 20.8% admitted they did not know the cause. Other misconceptions persisted: 17.0% thought it was caused by bacteria, 3.0% by protozoa, and 2.4% by fungi. Respondents were asked about rabies symptoms (multiple responses allowed) and the most commonly recognized symptoms were behavioral changes (46.6%) and seizures (45.3%). Other symptoms cited included fever (46.1%), hydrophobia (36.7%), inability to swallow (31.8%), paralysis (19.4%), dropped jaw (7.5%) and pica (11.9%); 21.6% responded “I do not know” to the symptom question. Most respondents understood that dog bites can transmit rabies – 81.4% identified dog bites as a mode of transmission. A majority (59.3%) also recognized that the virus can penetrate open wounds via dog saliva. All other listed transmission routes had lower recognition, and 9.2% reported “I do not know” for transmission routes. Regarding prevention of human infection from dogs, 58.8% of aware respondents identified human vaccination as a preventive measure, and 65.0% identified mass vaccination of dogs. A smaller proportion mentioned other measures, for example 35.6% suggested killing stray dogs and 32.6% incorrectly suggested antibiotics. Only 9.7% answered “do not know.” For control of rabies in the dog population, 65.0% recommended mass dog vaccination and 54.2% recommended public awareness campaigns. Other options – killing stray dogs (40.4%) and spaying (41.5%) – were cited less frequently, and 9.7% said “do not know” (Table 2). This indicates that although basic awareness of rabies was high, detailed knowledge of symptoms and control measures was limited in most participants. (Table 2).

**Table 2:** Awareness and knowledge of canine rabies among respondents in Palestine.

Variable	Response / Item	*n*	%
Have you heard of rabies? (*N* = 417)	Yes	371	89.0
	No	46	11.0
Cause of rabies (*n* = 371) *	Bacteria	63	17.0
	Fungi	9	2.4
	Protozoan	11	3.0
	Virus	211	56.9
	I do not know	77	20.8
Symptoms of rabies (multi-response, *n* = 371) *	Behavioral changes	173	46.6
	Dropped jaw	28	7.5
	Fever	171	46.1
	Hydrophobia	136	36.7
	I do not know	80	21.6
	Inability to swallow	118	31.8
	Paralysis	72	19.4
	Pica	44	11.9
	Seizures	168	45.3
Modes of transmission of rabies (multi-response, *n* = 371) *	Blood	74	19.9
	Direct contact	54	14.6
	Dog bites	302	81.4
	Penetration of open wound with dog saliva	220	59.3
	I do not know	34	9.2
Prevention of transmission from dogs to humans (multi-response, *n* = 371) *	Antibiotics	121	32.6
	Human vaccination	218	58.8
	Killing stray dogs	132	35.6
	Mass dog vaccination	204	55.0
	I do not know	36	9.7
Control of rabies in dogs (multi-response, *n* = 371) *	Public awareness campaigns	201	54.2
	Spaying	154	41.5
	Killing of stray dogs	150	40.4
	Mass dog vaccinations	241	65.0
	I do not know	36	9.7
Knowledge score (*n* = 371)	Mean (SD)	6.71	(3.54)
Satisfactory knowledge (*n* = 371)	≥50% score	157	42.3

* Multiple responses allowed. Percentages do not sum to 100.

### Practices related to rabies management and dog ownership

All 417 respondents answered the practice questions. However, only 20 respondents (4.8%) reported current dog ownership, while 397 (95.2%) did not own dogs. It is important to note that the practice results below represent a mix of actual behaviors (among the 20 dog owners) and hypothetical or intended responses (among the 397 non-owners) (Table 3). For example, non-owners were asked how they would respond if they owned a dog or experienced a dog bite, whereas dog owners reported their actual practices. This distinction should be considered when interpreting the findings, as hypothetical intentions may not fully align with actual behaviors. In the event of a dog-bite, 83.2% of respondents indicated they would take the victim to a hospital or health facility, and 11.8% said they would immediately wash the wound with soap and water. A small proportion reported other actions: 1.0% self-treat, 1.0% use traditional medicine, 0.5% take the victim to a spiritual healer, and 2.6% said they would do nothing.

**Table 3:** Practices regarding dog ownership and rabies prevention among respondents in Palestine (*N* = 417).

Variable	Response / Item	*n*	%
Dog ownership	Yes	20	4.8
	No	397	95.2
Action after dog-bite incident	Wash with soap and water immediately	49	11.8
	Take victim to hospital/health facility	347	83.2
	Take victim to spiritual healers	2	0.5
	I use traditional medicine	4	1.0
	I practice self-treatment	4	1.0
	Nothing	11	2.6
If you own a dog, would you allow a certified veterinarian or trained veterinary technician to vaccinate it?	Yes	400	95.9
	No	17	4.1
If you own a dog, do/would you typically confine it?	Always	162	38.8
	Sometimes	156	37.4
	Never	30	7.2
	I do not know	69	16.5
If you own a dog, who primarily do/would cares for it?	Children	6	1.4
	Everybody	238	57.1
	Father	163	39.1
	Mother	10	2.4
If you own a dog, what do you/would you typically feed it? *	Commercial dog feeds	268	64.3
	Cook special pot	61	14.6
	Family left-over	226	54.2
Regarding dog ownership, what is/would be the source of a dog for you?	Bought	35	8.4
	Gift	13	3.1
	Offspring of an owned dog	6	1.4
	Found / Rescued	16	3.8
	I do not know	19	4.6
	I do not plan to own a dog	328	78.7
If you own a dog, how is it typically managed when it can no longer be kept?	Give away for someone else	292	70.0
	I do not give out my dogs	39	9.4
	Sell them	86	20.6
Do you think veterinary services are adequate in your community?	Yes	31	7.4
	No	283	67.9
	Maybe	103	24.7

* Multiple responses allowed. Percentages do not sum to 100.

Almost all respondents (95.9%) stated they would allow a certified veterinarian or trained veterinary technician to vaccinate their dog, whereas 4.1% would not. Regarding dog confinement, 38.8% said they would always confine their dog, 37.4% sometimes confine it, 7.2% would never confine it, and 16.5% were unsure. If they owned a dog, 57.1% indicated “everybody” would primarily care for it, 39.1% said the father, and small minorities said the mother 2.4% or children 1.4% In terms of feeding, 64.3% reported they would feed commercial dog food, 54.2% family leftovers, and 14.6% would cook a special pot for the dog.

Among all respondents, 8.4% said they would obtain a dog by buying one, 3.8% by finding or rescuing one, 3.1% by gift, and 1.4% from offspring of an owned dog. The majority 78.7% stated they do not plan to own a dog. If a dog could no longer be kept, 70.0% of respondents said they would give it away to someone else, 20.6% said they would sell it, and 9.4% said they would not give it away. Finally, when asked about veterinary services in their community, only 7.4% felt services were adequate, whereas 67.9% felt they were inadequate and 24.7% were unsure.

### Factors associated with satisfactory rabies knowledge

Among the 371 respondents who had heard of rabies, multivariable logistic regression identified several demographic factors significantly associated with satisfactory knowledge. Age was inversely associated with knowledge: in multivariate analysis, those 36–45 years old had OR = 0.49 (95% CI: 0.24–0.99; *p* = 0.048) and those ≥46 years had OR = 0.35 (95% CI: 0.17–0.73; *p* = 0.005), each relative to 18–25-year-olds. Education again showed strong associations: medium education was associated with higher knowledge (OR = 2.86, 95% CI: 1.02–8.03; *p* = 0.046) and high education with even higher knowledge (OR = 4.66, 95% CI: 2.23–9.72; *p* < 0.001) versus low education. On univariate analysis, students had greater odds of satisfactory knowledge (OR = 1.75; *p* = 0.043) but this was not significant after adjustment. No significant associations were observed for gender, locality, or household economic status in the multivariate model. These results are detailed in Table 4.

**Table 4:** Univariate and multivariate logistic regression analysis of demographic variables associated with satisfactory rabies knowledge in Palestine (*N* = 371).

Outcome	Variable	Referent	Category	Univariate OR (95% CI)	*p*	Multivariate OR (95% CI)	*p*
Knowledge (*N* = 371)	Age group	18–25	Ref	Ref		Ref	
			26–35	0.59 (0.33–1.05)	0.071	0.51 (0.25–1.02)	0.058
			36–45	0.62 (0.36–1.09)	0.096	0.49 (0.24–0.99)	0.048 *
			≥46	0.38 (0.21–0.68)	0.001 ***	0.35 (0.17–0.73)	0.005 **
	Education	Low	Ref	Ref		Ref	
			Medium	2.26 (0.86–5.94)	0.100	2.86 (1.02–8.03)	0.046 *
			High	3.32 (1.72–6.39)	<0.001 ***	4.66 (2.23–9.72)	<0.001 ***
	Employment	Employed	Ref	Ref		Ref	
			Not working	1.00 (0.60–1.68)	0.996	1.06 (0.60–1.87)	0.849
			Student	1.75 (1.02–3.03)	0.043 *	1.43 (0.66–3.11)	0.363
	Locality	City	Ref	Ref		Ref	
			Village/Camp	1.06 (0.70–1.61)	0.774	1.05 (0.67–1.66)	0.822
	Economic	Low	Ref	Ref		Ref	
			Average	1.32 (0.76–2.30)	0.316	1.02 (0.56–1.85)	0.956
			High	1.35 (0.61–2.96)	0.461	0.83 (0.35–1.99)	0.678
	Gender	Male	Ref	Ref		Ref	
			Female	1.40 (0.92–2.12)	0.118	1.48 (0.93–2.35)	0.098

OR, Odds Ratio; CI, Confidence Interval.

Reference categories: Age 18–25, Low education, Employed, City, Low economic status, Male.

Knowledge outcome = satisfactory knowledge score, among those aware (*N* = 371).

Significance codes: * *p* < 0.05; ** *p* < 0.01; *** *p* < 0.001.

## Discussion

This cross-sectional study offers the first comprehensive examination of how Palestinian adults understand and respond to rabies risks. Although general awareness of rabies was widespread, the depth and accuracy of this awareness were limited. Several misconceptions emerged that have important implications for prevention efforts, including uncertainty about the viral cause of rabies and confusion between appropriate first-aid measures and ineffective alternatives. The gap between recognizing rabies as a serious disease and knowing the correct immediate response to a potential exposure highlights a critical weakness in community-level preparedness. Furthermore, many respondents expressed concerns about the adequacy of local veterinary services, suggesting that perceived infrastructural deficiencies may undermine confidence in prevention strategies and reduce engagement with formal health or veterinary systems. Taken together, these patterns indicate that while rabies is well-recognized as a health concern, essential biomedical concepts and preventive behaviors have not been fully internalized, leaving communities vulnerable to delays in seeking appropriate care.

The analysis of demographic patterns reveals important opportunities for targeted intervention. Younger adults and individuals with higher educational attainment demonstrated a stronger grasp of key preventive concepts, whereas older adults and those with more limited formal education appeared to have less accurate or less complete information. These differences underscore the need for tailored communication strategies that address specific knowledge gaps in different population groups. Interventions for older adults may require more foundational health education, whereas younger or more educated individuals may benefit from reinforcement of correct first-aid responses and guidance on navigating available services. The widespread perception that veterinary services are insufficient further suggests the need to strengthen public confidence through more visible, community-centered programs such as dog vaccination campaigns and municipal engagement. By addressing both the informational gaps and the systemic barriers identified in this study, public health authorities can support more consistent and effective rabies prevention behaviors across the Palestinian population.

The findings from this study are consistent with patterns observed in similar rabies knowledge, awareness, and practices surveys conducted in other endemic regions. In a recent study in Nepal, for instance, an online survey on rabies prevention found high general awareness but significant gaps in detailed knowledge and preventive practices (13). While 86.5% of respondents were aware of rabies, only 38.4% identified the virus as the causative agent, and only 27.8% recognized the correct preventive measures, such as timely post-exposure prophylaxis (PEP). These findings mirror the Palestinian context, where although 89.0% of respondents had heard of rabies, only 56.9% accurately identified a virus as the cause, and much confusion persisted around preventive measures like vaccination. The overlap in these results indicates that, despite widespread awareness, critical aspects of rabies control such as understanding its viral nature and the importance of PEP remain insufficiently communicated. This is especially evident in the substantial number of respondents endorsing antibiotics (32.6%) as a preventive measure. These misconceptions can lead to delays in seeking appropriate treatment, contributing to preventable deaths in rabies-endemic regions.

Recent KAP surveys in conflict-affected settings reveal persistent gaps despite high superficial awareness. For example, 85% of Kabul (Afghanistan) residents had heard of rabies, yet only 46% held substantive disease knowledge (e.g., half knew it was vaccine-preventable) (14). Similarly, in Iraq’s Kirkuk region 81% identified dogs as rabies vectors but a mere 10% could recognize a rabid animal (15). Notably, hospital treatment after dog bites was reported by ≈74% of Iraqi respondents (versus 83% in the Palestinian cohort) (15). In Palestine, by contrast, 89% awareness co-occurred with just 42% “satisfactory” knowledge and only ~12% performing proper wound washing, while ≈33% believed (incorrectly) that antibiotics could treat rabies. This pattern — high general awareness but poor concrete knowledge or practice — echoes findings from fragile contexts. For instance, a 2024 Sudanese physician survey (in a country beset by conflict) found only ~52% had adequate rabies knowledge (16). Likewise, in Yemen rabies remains endemic amid weak surveillance (≈30 reported cases/year, possibly ~220 actual, mostly from dog bites) (17), suggesting that disrupted health systems hinder prevention. Thus, although populations in war-affected regions may know of rabies, their understanding of prevention (wound care, vaccination) and care pathways remains limited (14, 15). These comparative data underline that even where awareness is high, conflict settings suffer critical knowledge/practice gaps and misconceptions, reinforcing the need for targeted education and One Health interventions in fragile settings.

Cultural beliefs and social norms in Palestine also play an important role in shaping rabies-related behaviors and may help explain some of the observed gaps in preventive practices. In many communities, dogs especially stray dogs are perceived with fear, suspicion, or religious sensitivity, which may discourage individuals from interacting with veterinary services or reporting animal bites. Traditional healing practices remain influential in some rural areas, where individuals may initially seek herbal remedies, spiritual treatments, or community advice rather than immediate medical care. This aligns with our finding that a small proportion of respondents preferred traditional or spiritual approaches following a bite. Social stigma associated with dog ownership, as well as misconceptions regarding the necessity of confining or vaccinating pets, may further limit engagement with preventive measures. Understanding these cultural and behavioral determinants is essential for designing targeted health education programs that are both culturally sensitive and aligned with community values.

Local municipalities also play a critical role in rabies prevention through their responsibility for managing stray dog populations. Municipal authorities oversee waste management systems, environmental sanitation, and community infrastructure all factors that influence the density and behavior of stray dogs. Implementing humane stray dog population control measures, such as coordinated vaccination campaigns, catch-neuter-vaccinate-release (CNVR) programs, and collaboration with veterinary services, can significantly reduce the number of unvaccinated free-roaming dogs. Municipal involvement is especially important in Palestine, where resource constraints and inter-governorate mobility challenges can limit centralized public health interventions. Strengthening municipal engagement, improving coordination with veterinary departments, and ensuring sustainable funding for stray dog management initiatives would therefore enhance rabies control efforts and reduce transmission risks at the community level.

Further evidence from Nigeria’s Gombe State supports these findings, where rabies awareness was similarly high (85%) but key knowledge gaps persisted, particularly regarding modes of transmission and post-exposure care (18). In contrast to the 83.2% of participants in Palestine who would seek professional medical attention after a dog bite, practices surrounding first-aid interventions like wound washing were markedly lower (11.8%). In Nigeria, only 39% of respondents were aware of the need for immediate medical care following an animal bite, further underscoring the critical need for early interventions. Both studies highlight the need for targeted educational campaigns focusing on the most effective first-aid actions, particularly wound irrigation, which is universally recognized as a key step in reducing rabies transmission risk.

Additionally, a systematic review of rabies vaccination programs across the Arabian Gulf region found a pattern of inadequate access to rabies vaccines and inconsistent post-exposure care, which is consistent with our finding that 67.9% of Palestinian respondents perceived local veterinary services as inadequate (19). This perception of limited access may contribute to reluctance in seeking timely PEP and undermine efforts to maintain high levels of dog vaccination. Studies from the Gulf region also emphasize the importance of integrated, community-based approaches to both animal and human health, aligning with the One Health approach endorsed by the World Health Organization (WHO). This highlights the need for improvements in veterinary service access, alongside public health education that addresses not only the scientific understanding of rabies but also practical service availability. In summary, our findings are consistent with those from other endemic regions, confirming that while rabies awareness is high, significant gaps remain in understanding key concepts like transmission, prevention, and appropriate first-aid. These results emphasize the need for targeted, region-specific interventions that integrate both public education and system-level improvements in healthcare and veterinary services, in line with the One Health approach.

The analysis of demographic determinants in this study reveals significant associations between age, education, and both rabies awareness and knowledge. As observed in previous studies, older respondents were more likely to have heard of rabies, but were less likely to exhibit satisfactory knowledge compared to their younger counterparts (20). This finding may reflect differences in educational exposure or updated knowledge regarding rabies control, which is often more prevalent in younger, more educated populations. Conversely, education emerged as a key predictor of rabies knowledge, with respondents holding higher levels of education demonstrating significantly better understanding of the disease (21). This highlights the importance of literacy and access to health education in shaping community understanding of zoonotic diseases like rabies.

These patterns suggest that educational interventions should be tailored to address specific demographic groups. For example, younger, more educated individuals may benefit from in-depth, guideline-driven messaging that emphasizes practical actions, such as wound washing and timely presentation for post-exposure prophylaxis (PEP) (22). In contrast, older adults, who may have more limited access to recent health information, might require more foundational education about rabies, its transmission routes, and the importance of early medical intervention. Given that formal education correlates strongly with better knowledge, community-based educational campaigns should prioritize improving literacy around rabies and animal health, particularly in rural areas where educational resources may be scarcer.

The perception of inadequate veterinary services, reported by 67.9% of respondents, also underscores a significant barrier to effective rabies prevention. This finding is consistent with other studies in rabies-endemic regions, which show that lack of access to veterinary care can hinder mass dog vaccination efforts and reduce the public’s willingness to engage with rabies control measures. For instance, in regions where veterinary services are perceived as insufficient, individuals may be less likely to vaccinate their pets or seek timely post-exposure care after a bite. To address this, local governments and health authorities should prioritize improving the accessibility and availability of veterinary services, especially in underserved areas. In addition to this, public health campaigns should not only focus on educating the population about rabies but also aim to reinforce the importance of veterinary services and their role in both animal and public health.

Furthermore, the perceived inadequacy of veterinary care highlights the need for stronger community-based surveillance systems, which can serve as early warning systems for rabies outbreaks and facilitate timely interventions. The integration of human and animal health surveillance through a One Health approach is essential for improving rabies control strategies (23). By enhancing the capacity for both human and veterinary healthcare providers to detect, report, and respond to rabies cases, this approach can reduce the risk of transmission and ensure that preventive measures, such as vaccination and post-exposure prophylaxis, are more widely implemented and accessible (24). Addressing the educational gaps and improving access to veterinary care are critical steps for enhancing rabies control in Palestine. By focusing on the identified determinants age, education, and service access public health programs can more effectively tailor their interventions to the needs of different population groups. These efforts, coupled with strengthened veterinary infrastructure and community engagement, will be essential in reducing rabies incidence and moving toward the eventual elimination of the disease (25).

Several countries with resource-limited settings have implemented successful rabies prevention programs that offer valuable models for Palestine. Tanzania, for example, achieved substantial reductions in human rabies deaths through annual mass dog vaccination campaigns combined with decentralized access to PEP and community-level surveillance systems (26). In Nepal, community education campaigns delivered through schools and municipal health departments significantly improved public understanding of wound care and reduced delays in seeking PEP (13). The Philippines provides another strong example, where integrated One Health approaches, strong municipal involvement, and mobile vaccination units successfully expanded dog vaccination coverage and improved reporting of bite incidents (27). Palestine can adopt or adapt these approaches by implementing coordinated mass dog vaccination drives, strengthening collaboration between public health, veterinary, and municipal authorities, and deploying mobile vaccination and awareness units to reach underserved rural areas. Adapting global best practices to the Palestinian context particularly through local governance structures could greatly enhance the effectiveness of rabies control efforts.

### Recommendation

The findings of this study carry several important policy implications for public health authorities in Palestine. Strengthening national mass dog vaccination programs is essential, given the central role of dog-mediated transmission and the widespread public perception of inadequate veterinary services. Expanding dog vaccination coverage particularly in rural areas requires improved veterinary workforce capacity, mobile vaccination campaigns, and coordinated partnerships with municipalities and local NGOs. Likewise, access to timely post-exposure prophylaxis (PEP) must be improved through decentralizing availability to primary healthcare centers, ensuring continuous vaccine supply chains, and training frontline health workers in standardized rabies management protocols. Enhancing rabies surveillance systems, integrating human and animal health reporting, and promoting public awareness of PEP availability are also critical steps. Taken together, these policy actions would substantially strengthen Palestine’s capacity to prevent rabies and reduce reliance on personal knowledge or informal practices after animal bites.

This study has several notable strengths. It represents the first large web-based survey on rabies awareness, knowledge, and practices in Palestine, providing important preliminary insights to guide future public health initiatives. The use of a validated, pre-tested questionnaire adapted from previous studies enhances the reliability of the findings and facilitates meaningful comparison with research conducted in other rabies-endemic regions. Although derived from a non-probability, web-based convenience sample, the large number of respondents improves the precision of estimates within this digitally connected population. The inclusion of diverse sociodemographic variables such as age, education, and geographic location allows for a nuanced description of factors associated with rabies-related knowledge and practices. Finally, ethical rigor was maintained throughout the study, with informed consent obtained from all participants and ethical approval provided by Al-Quds University.

### Limitation

Despite its strengths, this study has several limitations. First, the cross-sectional design limits our ability to draw causal inferences, as it only captures a snapshot of rabies knowledge and practices at one point in time. Second, the online snowball sampling method may have introduced selection bias, as it likely overrepresented younger, more educated, and digitally connected individuals while underrepresenting older or rural populations who may have limited internet access. This limits the generalizability of the findings to the broader Palestinian population. Third, self-reported data is prone to social desirability bias, particularly regarding health-related behaviors, as participants may have overstated their intentions to seek medical care or vaccinate their dogs. Finally, the lack of direct observation of actual behavior (such as seeking care post-bite) limits the ability to confirm whether respondents’ reported practices align with their real-world actions.

### Future research

To build upon the findings of this study, longitudinal research should be conducted to track changes in rabies knowledge and practices over time, particularly after the implementation of targeted educational interventions. Randomized controlled trials or intervention studies could be used to evaluate the effectiveness of different educational campaigns aimed at improving knowledge of rabies transmission, prevention, and first-aid practices. Additionally, future studies should consider using more representative sampling methods, such as random household surveys, to ensure that all demographic groups, especially those without access to the internet, are adequately represented. Further research into the barriers to seeking veterinary care, including both logistical and attitudinal factors, would help to better understand how to improve access to rabies prevention services. Geographically expanded studies across rural and underserved areas of Palestine would provide a more comprehensive picture of regional differences in rabies knowledge and prevention efforts. Lastly, canine morbidity and rabies surveillance are critical to better understanding the actual burden of rabies risk in Palestine, and studies that track dog bite incidents and veterinary reports of rabies will help to refine control strategies.

## Conclusion

This study provides essential insights into rabies knowledge and practices in Palestine, revealing high general awareness but significant gaps in understanding critical aspects such as viral etiology, transmission, and preventive measures, particularly post-exposure prophylaxis and immediate wound care. Sociodemographic factors, including age and education, were key determinants of rabies knowledge, highlighting the need for tailored educational interventions. Additionally, the perceived inadequacy of veterinary services presents a barrier to effective rabies prevention. To improve public health outcomes, it is crucial to address these knowledge gaps through targeted awareness campaigns, emphasize the importance of wound care and timely medical intervention, and enhance access to veterinary services.

Based on these findings, several practical health education strategies are recommended to improve rabies prevention in Palestine. Workshops delivered through municipalities, primary healthcare centers, and local organizations could help disseminate accurate information about rabies transmission, wound care, and the urgency of post-exposure prophylaxis. School-based awareness programs, including curriculum modules, posters, and interactive activities, would be valuable for educating children and adolescents, who are among the most vulnerable groups for dog bites. Veterinary outreach campaigns particularly mobile clinics and community vaccination drives could address the widely perceived inadequacy of veterinary services and improve access to dog vaccination in rural and underserved areas. Tailored media campaigns using social media, radio, and community influencers may also help dispel misconceptions and promote timely care-seeking behaviors. Implementing these targeted educational interventions would enhance community preparedness and contribute to more effective rabies control efforts across Palestine. A comprehensive One Health approach, integrating human, animal, and environmental health, is essential for the successful control and eventual elimination of rabies in Palestine.

## Data Availability

The original contributions presented in the study are included in the article/supplementary material, further inquiries can be directed to the corresponding author.
